# Knowledge, attitudes and practices of malaria transmission and preventive measures in Woreta town, Northwest Ethiopia

**DOI:** 10.1186/s13104-018-3607-z

**Published:** 2018-07-18

**Authors:** Amir Alelign, Beyene Petros

**Affiliations:** 1 Department of Microbial, Cellular and Molecular Biology, Addis Ababa University, P.O. Box 1176, Addis Ababa, Ethiopia; 2 Department of Biology, Debrebirhan University, P.O. Box 445, Debrebirhan, Ethiopia

**Keywords:** Malaria, Awareness, Woreta, Ethiopia

## Abstract

**Objective:**

Despite a high public health burden of malaria in endemic regions of Ethiopia, there are limitations on the availability of data concerning public awareness about the disease and its preventive measures. The present study aimed in producing base line data on the community knowledge, attitudes and practices towards malaria transmission and its preventive measures in Woreta town, northwest Ethiopia. A community based two-stage random cluster study was conducted from May to July 2013. Household heads were interviewed to assess their awareness about malaria and its control measures.

**Results:**

About 78.5% (113/144) of the respondents rated bite of infected mosquito as a way of malaria transmission. The majority of participants, 126 (87.5%) stated one or more symptoms of malaria. About 95.8% (138/144) of the respondents indicated that malaria is preventable and curable disease. Only about 25% (36/144) of the study participants practiced frequent and proper use of insecticide treated bed nets (ITNs). Draining logged water was a highly rated, 83 (57.6%), practice of environmental management of malaria.

**Electronic supplementary material:**

The online version of this article (10.1186/s13104-018-3607-z) contains supplementary material, which is available to authorized users.

## Introduction

Malaria remained to be one of the most sever public health problem worldwide, particularly, in poor tropical and subtropical areas. In 2016, an estimated 445,000 people died of malaria, most were young children in sub-Saharan Africa [1]. Despite the implementation of preventive and control efforts against malaria for decades, it remained to be the leading communicable disease seen at health facilities in Ethiopia [2]. The major intervention strategies that were applied in the country to combat malaria were: early diagnosis and prompt treatment, selective vector control that involves use of indoor residual spraying (IRS), insecticide-treated mosquito nets (ITNs) and environmental management [3]. However, all these efforts were not based on active participation and knowhow of the local community. Hence, an integrated effort that actively involves the local communities with their full knowledge is essential to enhance the disease controlling program in malaria endemic regions of the country.

From different parts of Africa, including Ethiopia, considerable number of community-based studies were conducted about the level of awareness about malaria and associated risk factors to its transmission and control measures [4–13]. These studies concluded that misconceptions concerning malaria still exist and that practices for the control of malaria have been unsatisfactory. Therefore, enhancing awareness, beliefs and practices of the community with respect to the disease as well as the identification of special factors contributing to its transmission is required. Thus, the present study aimed to collect baseline information concerning knowledge, attitudes and practices of people in Woreta town, northwest Ethiopia regarding malaria and its preventive measures. Moreover, the study assessed the associated risk factors to the transmission of the disease.

## Main text

### Methods

#### Study design and population

A community based two-stage random cluster study was conducted from May to July 2013 at Woreta town. Woreta is known for its flat and low land regarded as malarious area. Malaria was the most prevalent seasonal disease in the area, and October to December was the peak transmission season [14]. A total of 144 households were selected using probability proportion to size of households in four ‘Kebeles’ (the smallest administrative units) of the town. The shared households for each Kebeles were divided by the total number of households in a given kebele to determine a sampling interval for selecting households. Accordingly, every 15th households was selected using systematic random sampling technique [4]. The study population included those aged 15 years and above of both sexes.

#### Data collection

A structured questionnaire was designed and administered by data collectors (Additional file 1). The questioner was first developed in English and then translated into ‘Amharic’, the local language. The household heads were interviewed on their awareness, attitudes and practices towards malaria transmission and its preventive methods. A person who had been thought as the primary decision maker in the family was considered as the head of the household. Whereas, a household was defined as a group of individuals living together in a house and sharing common facilities [15]. In case of the household head absence, a responsible adult above 15 years old who was appointed by the family was interviewed.

#### Data quality control

Before starting the actual work, the quality of the questioners was checked for serial number, quantity and procedure of collection. Pre-test was conducted on 5% of the sample size at each ‘kebele’ to ensure the validity of the data collection and to standardize the questionnaire. The first author checked the questionnaires for completeness every day. Incomplete questionnaires were returned to data collectors for correction by revisiting the households.

#### Data analysis

The data were double entered in Microsoft Excel data sheets and analyzed using SPSS 16 software. Descriptive statistics were carried out to measure relative frequencies, percentages, averages, and relative frequencies of the variables. The risk factor analysis to the number of malaria illness and repeated exposure was done using univariate analysis at 95% confidence interval (CI) for odds ratio and p-values < 0.05 were considered to be statistically significant.

### Results

#### Knowledge, attitude and practices of household respondents towards malaria

About 68.5% (99/144) of the respondents stated mosquito as cause of malaria and only 24.3% (35/144) of them rated plasmodium parasite as cause of malaria. Drinking unsafe water and eating contaminated food were also considered to be means of malaria transmission by 11.1% (16/144) and 6.9% (10/144) of the respondents, respectively. Bite of infected mosquito was most rated, 113 (78.5%) as a way of malaria transmission (Table 1). The majority of respondents, 126 (87.5%) stated one or more symptoms of malaria and fever was the most frequently stated, 49 (34%) characteristic followed by shivering, 36 (25%). Only about 12.5% (18/144) of the respondents did not know how malaria was characterized.

**Table 1:** Knowledge and attitudes of respondents related to the cause, transmission and symptoms of malaria (N = 144), Woreta town, Northwest Ethiopia, 2013

Characteristics	Categories	Frequency (%)
Cause of malaria	Mosquitoes	99 (68.5)
	Plasmodium	35 (24.3)
	Worms	8 (5.6)
	Unknown	2 (1.4)
Ways of malaria transmission	Bite of mosquitoes	113 (78.5)
	Unsafe water	16 (11.1)
	Contaminated food	10 (6.9)
	Personal contact	5 (1.1)
Symptoms of malaria	Fever	49 (34.0)
	Shivering	36 (25.0)
	Sweating	21 (4.6)
	Head ache	20 (13.9)
	Unknown	18 (12.5)

N = total number of respondents

About 95.8% (138/144) of the respondents stated malaria as a preventable and curable disease and 120 (83.4%) of them had encountered malaria illness repeatedly for two or more times in their life time. All of the respondents replied that they possessed at least one insecticide-treated mosquito net (ITN) in their houses. Out of which, only 36 (25%) of them used ITNs frequently in their house to prevent mosquito bite. The three main components of the environmental management activities in the control of malaria vector were draining logged water, environmental clearing and educating other people to create awareness on malaria and its control methods. Of which, draining logged water was the activity most respondents, 83 (57.6%) were participated (Table 2).

**Table 2:** Practices and attitudes of respondents towards control and preventive measures of malaria, Woreta town, Northwest Ethiopia, 2013

Characteristics	Categories	Frequency (%)
Malaria is preventable and curable	Yes	138 (95.8)
	No	6 (4.2)
Type of antimalarial drug used	Chloroquine	48 (33.3)
	Coartem^®^	68 (47.2)
	Other antimalarials	28 (19.4)
Use of at least one ITNs in the house	Yes	144 (100)
	No	0 (0)
Trend in the use of ITNs	Always	36 (25.0)
	Sometimes	98 (68.1)
	Do not use	10 (6.9)
Practice in malaria control activities	Draining logged water	83 (57.6)
	Environmental clearing	39 (27.1)
	Educating people	22 (15.3)

#### Risk-factor analysis for malaria illnesses

Although there were no significant differences between the risk factors to the previous illnesses of malaria, it was comparable among some of the different factors (Table 3). The odds of previous malaria illness was lower among communities within age groups 30–45 years than those of above 29 years (crude odds ratio [COR]; 0.78, 95% confidence interval [CI] 0.251–2.468). Similarly, the chance of having previous malaria illnesses was lower in government employees when compared to those engaged in private businesses (COR; 0.39, 95% CI 0.1014–1.492). No comparable differences in their previous malaria exposure had been noticed between males and females in the community (COR; 1.04, 95% CI 0.4095–2.634). However, the odds of previous malaria illnesses were higher in non-educated individuals than those who had formal or informal education (COR; 1.59, 95% CI 0.5036–5.042). In this analysis, considering religion as a risk factor for the previous malaria illness, Muslims had higher odds than Orthodox Christians (COR; 1.57, 95% CI 0.5183–4.745) (Table 3).

**Table 3:** The distribution of some selected socio-demographic risk factors univariate analysis for previous illness of malaria among respondents, Woreta town, Northwest Ethiopia, 2013

Variables	Categories	Frequency (%)	Independent effects			p-value
			House hold respondents (N = 144)			
			Yes	No	Crude OR	
			n (%)	n (%)	(95% CI)	
Age (years)	15–29^a^	32 (22.2)	27 (84.4)	5 (15.6)	1.00	0.84
	30–45	63 (43.8)	51 (81.0)	12 (19.0)	0.78 (0.251–2.468)	
	46–60	33 (22.9)	29 (87.9)	4 (12.1)	1.34 (0.326, 5.529)	
	> 60	16 (11.1)	13 (81.3)	3 (18.7)	0.80 (0.1658, 3.883)	
Educational status	Educated^a^	111 (77.1)	91 (82.0)	20 (18.0)	1.00	0.44
	Non-educated	33 (22.9)	29 (87.9)	4 (12.1)	1.59 (0.5036, 5.042)	
Sex	Male^a^	47 (32.6)	39 (83.0)	8 (17.0)	1.00	0.92
	Female	97 (67.4)	81 (83.5)	16 (16.5)	1.04 (0.4095, 2.634)	
Occupation	Private business^a^	43 (29.9)	36 (83.7)	7 (16.3)	1.00	0.32
	GO employee	15 (10.4)	10 (66.7)	5 (33.3)	0.39 (0.1014, 1.492)	
	Daily laborer	29 (20.1)	25 (86.2)	4 (13.8)	1.22 (0.3214, 4.596)	
	House wife	57 (39.6)	49 (86.0)	8 (14.0)	1.19 (0.3957, 3.584)	
Religion	Orthodox^a^	76 (52.8)	63 (82.9)	13 (17.1)	1.00	0.41
	Muslim	43 (29.9)	38 (88.4)	5 (11.6)	1.57 (0.5183, 4.745)	
	Protestant	25 (17.4)	19 (76.0)	6 (24.0)	0.65 (0.2186, 1.953)	

*n* number of respondents for the category, *OR* odds ratio

^a^Reference category

### Discussion

The findings of this study showed that general awareness about malaria and its control methods was high among Woreta town communities. About 78.5% of the study participants associated mosquito bite to malaria. This was a little higher than a study reported in Jimma town (71.8%), the other part of Ethiopia [16]. About 24% of the respondents were aware of plasmodium parasites as the cause of malaria. The possible explanation about the poor knowledge on the cause of malaria could be misconceptions about causes and ways of transmission of malaria. Their previous knowledge in associating malaria with mosquito bite might have influenced some members of the community. Misconceptions about cause of malaria were also reported in other similar studies [17, 18].

The knowledge of respondents whether malaria is preventable and curable was very high (95.8%). In agreement, other studies conducted in Ethiopia reported higher knowledge of communities on malaria as preventable and curability [4, 19]. About 57.6 and 27.1% of the respondents were engaged in malaria control activities in the community through draining logged water and environmental clearing, respectively. The observed participation in control activities was lower than reports from studies of other areas in Ethiopia, 73% in town and 26.9% in rural areas [8, 20]. Such differences in the practice of managing malaria might be due to differences in awareness on the transmission methods of the disease and differences in the leadership efforts to mobilize communities to participate in the malaria control activities among the different study areas.

All the respondents possessed at least one ITN in their house. However, the majority of them (68.1%) were not frequently using the bed nets. Despite the availability of the nets, misuse of them was noticed as it had been observed in other studies in some parts of Africa [21–23]. Failure in the use of possessed nets among the community might have been due to lack of awareness on its proper use and its effectiveness in preventing mosquito bite.

Previous reports suggested that malaria prevalence had been influenced by other socio-demographic factors [24, 25]. In our study, there were comparable differences within some of the socio-demographic characteristics such as age, educational status, occupation, sex and religion of the respondents; however, no significant difference was observed. This lack of significant difference might have been due to the fact that some other potential factors such as mismanagement of the ongoing massive rice irrigation activity might have contributed for the occurrence of the disease in the study area.

### Conclusion

Overall, awareness about malaria and its control measures was high among Woreta town community. Despite this, occasional use of ITNs was observed to be one of the major setbacks for the effort to control the decrease. Therefore, awareness campaign should be strengthened in this respect.

## Limitation

The present study was limited in assessing the influence of other factors such as nutritional status and indications of co-infections which may have contributed for the occurrence of the disease.

## Additional file

**Additional file 1.** Semi-structured questionnaire for respondents of Woreta town, Fogera district, northwest Ethiopia: the questionnaire was designed to collect data on knowledge, attitudes and practices on malaria transmission and control measures. It contained three components; (a) instruction to the respondents, (b) socio-demographic characteristics of respondents and (c) participants’ knowledge, attitudes and practices on malaria transmission and its prevention.

## Abbreviations

CI: confidence interval
CQ: chloroquine
DDT: dichloro diphenyle trichloro ethane
HHs: house holds
IRS: indoor residual spraying
ITNs: insecticide-treated mosquito nets
KAPs: knowledge, attitudes and practices
OR: odds ratio
SPSS: statistical package for the social sciences
