# Supplementary material for: Knowledge, attitudes and practices of malaria transmission and preventive measures in Woreta town, Northwest Ethiopia
Source: BMC Res Notes. 2018 Jul 18;11:491. doi: 10.1186/s13104-018-3607-z (PMC6052557; doi:10.1186/s13104-018-3607-z)
Supplement: Supplementary file 1 — Additional file 1. Semi-structured questionnaire for respondents of Woreta town, Fogera district, northwest Ethiopia: the questionnaire was designed to collect data on knowledge, attitudes and practices on malaria transmission and control measures. It contained three components; (a) instruction to the respondents, (b) socio-demographic characteristics of respondents and (c) participants’ knowledge, attitudes and practices on malaria transmission and its prevention. [file 13104_2018_3607_MOESM1_ESM.docx]

## Additional file 1

## Semi-structured questionnaire for respondents of Woreta town, Fogera District: to collect data on knowledge, attitudes and practices on malaria transmission and control measures

**A. Instruction to the respondents**

The purpose of this questionnaire is to obtain relevant information to conduct a research on topic “*Knowledge, attitudes and practices of malaria transmission and preventive measures in Woreta town, Northwest Ethiopia*”. So, your participation is very important for the realization of the study. Please be sure that the entire questionnaire is for the research purpose only and treated with at most confident. Therefore, as much as possible, please give the necessary information freely and honestly.

Please write your answers in the space provided and putting tick mark (√) on the Yes/No box provided.

**Thank you in advance for your participation.**

**B. Socio demographic characteristics of respondents**

Name _________________________________

Age ________

Sex__________________

Occupation_______________________ Religion____________________

Address____________________ No. of years living in the area_________

Migrated from________________________________

Education ________________________Specify________________

Language known_________________________

Incidents on malaria illness ______________times

**C. Participants’ knowledge, attitudes and practices on malaria transmission and its        prevention.**

| **No.** | **Questions** | **Yes** |  | **No** |
| --- | --- | --- | --- | --- |
| 1. | Have you got ill with malaria? |  |  |  |
| 2. | What do you think is the cause of malaria? |  |  |  |
|  | a. Parasitic micro organisms |  |  |  |
|  | b. Worms |  |  |  |
|  | c. Insects |  |  |  |
|  | d. Any other, if possible please mention **-------------------------------------         ---------------------------------------------------** |  |  |  |
| 3. | Do you know how the disease is transmitted?  If yes, in which way? |  |  |  |
|  | 1. Drinking unsafe water |  |  |  |
|  | b. Eating contaminated food |  |  |  |
|  | 1. Bite of infected mosquito |  |  |  |
|  | d. Contact with infected person |  |  |  |
|  | e. Any other**,----------------------------------------------------------                    ------------------------------------------------------------** |  |  |  |
| 4 | Do you know some symptoms experienced by a person diseased with malaria? If yes, which ones are the symptoms? |  |  |  |
|  | a. Fever |  |  |  |
|  | b. Sweating |  |  |  |
|  | c. Shivering |  |  |  |
|  | d.Head ache |  |  |  |
|  | e. Lack of sense of wellbeing |  |  |  |
|  | f. Any other**,------------------------------------------------------------                  ---------------------------------------------------------------** |  |  |  |
| 5. | Do you think that malaria is preventable and curable? |  |  |  |
| 6  . | Can you mention some ways to prevent malaria?  If yes, please mention some of them**---------------------------------------------------------------------------------------------------------------------------------------------------------------------------------------------------------------------------------------------------------------------------------------------------------------------------------------------------** |  |  |  |
| 7. | How do you manage yourself when you feel malaria illness? |  |  |  |
|  | a. Visit health centers |  |  |  |
|  | b. Self treatment, please mention if possible **----------------------------------------------------------------------------------------** |  |  |  |
| 8. | Do you know the importance of insecticide treated bed nets (ITNs) in preventing malaria? |  |  |  |
| 9. | Do you use ITNs in your house? |  |  |  |
| 10. | If yes, at which particular season you use ITNs? |  |  |  |
|  | 1. September---November |  |  |  |
|  | 1. December---February |  |  |  |
|  | 1. March---May |  |  |  |
|  | d. June---July |  |  |  |
|  | 1. Throughout the year |  |  |  |
| 11. | Where do you get ITNs? |  |  |  |
|  | 1. Health centers |  |  |  |
|  | 1. Drug stores |  |  |  |
|  | 1. Local shops |  |  |  |
| 12. | How is the trend in the accessibility of ITNs? |  |  |  |
|  | 1. Increased |  |  |  |
|  | 1. Decreased |  |  |  |
|  | 1. Remains the same |  |  |  |
| 13. | Do you participate in any malaria control activities in the community ? If yes in which way? |  |  |  |
|  | 1. Draining logged water |  |  |  |
|  | 1. Environmental cleaning |  |  |  |
|  | 1. Educating other people |  |  |  |
|  | 1. Any other way**----------------------------------------------------    ------------------------------------------------------------------** |  |  |  |
| 14. | Any other comment on malaria, preventing and controlling of the disease**-------------------------------------------------------------------------------------------------------------------------------------------------------------------------------------------------------------------------** |  |  |  |

##

## Thank you!
